# Solvent-free protic liquid enabling batteries operation at an ultra-wide temperature range

**DOI:** 10.1038/s41467-022-33612-2

**Published:** 2022-10-13

**Authors:** Mochou Liao, Xiao Ji, Yongjie Cao, Jie Xu, Xuan Qiu, Yihua Xie, Fei Wang, Chunsheng Wang, Yongyao Xia

**Affiliations:** 1 Department of Chemistry, Department of Materials Science, Shanghai Key Laboratory of Molecular Catalysis and Innovative Materials, Fudan University, Shanghai, 200433 China; 2 Department of Chemical and Biomolecular Engineering, University of Maryland, College Park, MD 20742 US; 3 College of Chemistry and Molecular Engineering, Zhengzhou University, Zhengzhou, 450001 China

**Keywords:** Batteries, Batteries, Batteries

## Abstract

Nowadays, electrolytes for commercial batteries are mostly liquid solutions composed of solvent and salt to migrate the ions. However, solvents of the electrolyte bring several inherent limitations, either the electrochemical window, working temperature, volatility or flammability. Herein, we report polyphosphoric acid as a solvent-free protic liquid electrolyte, which excludes the demerits of solvent and exhibits unprecedented superiorities, including nonflammability, wider electrochemical stability window (>2.5 V) than aqueous electrolyte, low volatility and wide working temperature range (>400 °C). The proton conductive electrolyte enables MoO_3_/LiVPO_4_F rocking-chair battery to operate well in a wide temperature range from 0 °C to 250 °C and deliver a high power density of 4975 W kg^−1^ at a high temperature of 100 °C. The solvent-free electrolyte could provide a viable route for the stable and safe batteries working under harsh conditions, opening up a route towards designing wide-temperature electrolytes.

## Abstract (web summary)

It is challenging to prepare electrolyte that could achieve wide electrochemical window, broad working temperature, non-inflammability, and fast ion transport simultaneously. Here the authors report a rocking-chair proton battery utilizing a solvent-free protic liquid electrolyte, which could operate in a broad temperature range from 0 to 250 celsius degree.

## Introduction

The tremendous demands for energy storage systems, especially rechargeable batteries with high safety, wide operating-temperature, and long cycle life, have aroused researcher’s great enthusiasm. Rechargeable batteries with liquid electrolytes have been developed and employed in portable electronics and electric vehicles^1–5^. Inside the batteries, electrolytes provide the ionic conductivity and allow for charge compensation. Conventional electrolytes are typically prepared by dissolving salts into solvents (aqueous or non-aqueous) to create solvated ions as charge carries^5–7^. The solvated ions facilitate the ion-transfer between electrodes and through the interface^8–12^. Besides that, the decomposition of solvation structure creates the solid electrolyte interphase (SEI) to enable the operation of electrodes beyond the thermodynamic stability window of the electrolyte^13,14^.

In spite of the merits, the traditional solvents in electrolytes also bring several limitations. The non-aqueous electrolytes normally contain highly toxic and flammable solvents, raising safety concerns of lithium-ion batteries(LIBs)^15^.The boiling and freezing points of solvents also limit the operation temperature range of LIBs^16,17^. Replacing the organic solvent with H_2_O could resolve the safety concern, while the narrow window of H_2_O limits the operation voltage and thus the energy density of aqueous batteries^18,19^. Water in salt (WIS) electrolytes gained a lot of attention in recent years for its extended stability window than the diluted aqueous electrolytes^20,21^. However, WIS electrolytes could not completely avoid the negative effects of solvents due to the presence of slight water, such as electrode dissolution and limited working temperature range, and the stability window of WIS is still limited by the decomposition of H_2_O. Besides the safety and the electrochemical window, the cyclability of batteries may also be influenced by the active materials dissolution in the solvent, as revealed by the shuttle problem of lithium polysulfides in the Li-S batteries^22,23^ and the inevitable dissolution of transition metal cation of lithium metal oxides cathodes^24^ in carbonate electrolytes. Neither organic nor aqueous electrolytes could fully meet the demand for high safety, wide temperature range, expanded electrochemical window, and stable cycling at the same time. Therefore, development of solvent-free electrolyte is critical for next generation of high performance batteries.

To remove the limitations brought by solvents, solid-state electrolytes (SSE) was proposed and has been receiving intense attentions. However, the poor contact between the rigid SSE and electrodes brings a series of interfacial issues, including high resistance, poor wettability, and mechanical instability^25,26^. Ionic liquids seem to be a compromise that possesses both mechanical stiffness and nonflammability. However, most typical ionic liquids that are fluxible at room temperature consist of relatively bulky cations and anions, which reduce ion transport rate and constrain reaction kinetic^6,27,28^.

Herein, we demonstrated the feasibility of the solvent-free protic liquid using polyphosphoric acid (denoted as PPA hereafter) as the electrolyte. PPA, with a molecular formula of H_n+2_P_n_O_3n+1_, is a liquid polymer formed by dehydration of phosphoric acid molecules^29^. Unlike typical protic ionic liquids that have an available proton on the big cation^30^, the cation of PPA is solely proton (H^+^), which has the smallest diameter and possesses rather fast migration speed. Therefore, PPA can be directly used as electrolyte in proton battery without solvents. Owing to the solvent-free characteristic of PPA, the electrode dissolution problem is mitigated and the electrochemical window is significantly expanded from 1.23 V (H_2_O) to over 2.5 V. The anodic limit of this electrolyte is beyond 2.0 V vs AgCl/Ag, which has never been achieved in any liquid electrolyte before^21,31^. Furthermore, PPA has the potential to work at very high temperature over 400 °C due to its high boiling point and noninflammability. A non-aqueous proton battery integrated with MoO_3_ anode and LiVPO_4_F cathode was constructed^32^. The solvent-free PPA shows excellent compatibility with both metal oxides anode and polyanion cathode, thus enabling the stable cycling of these electrodes, which otherwise decline rapidly in aqueous acid electrolytes. Surprisingly, the proton battery can even operate well in a wide temperature range from 0 °C to 250 °C and deliver a high capacity retention of 32% at the high rate of 100 C and 100 °C, superior than most of the liquid batteries reported so far.

## Results

### PPA and aqueous H_3_PO_4_ electrolytes

**Fig. 1:**
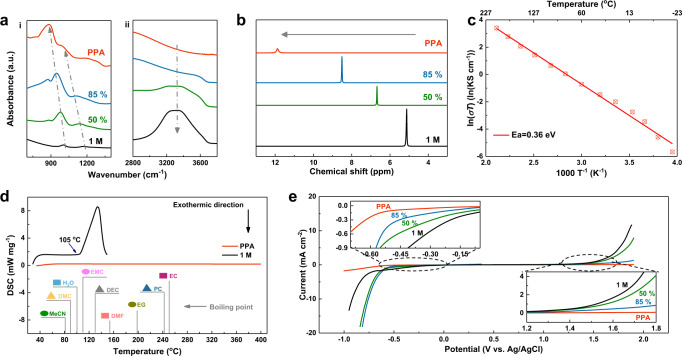
The comparison of various H_3_PO_4_ electrolytes. **a** ATR-FTIR and **b** ^1^H NMR spectra of 1 M H_3_PO_4_, 50 wt% H_3_PO_4_, 85 wt% H_3_PO_4_ and PPA. **c** The Arrhenius plot of PPA electrolyte. **d** DSC of the 1 M H_3_PO_4_ and PPA electrolyte. The boiling points of various solvents are also marked in the graph. **e** Electrochemical stability window of 1 M H_3_PO_4_, 50 wt% H_3_PO_4_, 85 wt% H_3_PO_4_, and PPA electrolytes measured by LSV on Ti mesh at a scan rate of 1 mV s^−1^.

The absence of solvent deviates the liquid structure of electrolytes. To demonstrate its unique physical and chemical properties, the solvent-free PPA was compared with phosphoric acid aqueous solutions with various concentrations. As shown in Fig. 1a, the attenuated total reflection Fourier transform infrared (ATR-FTIR) spectroscopy shows typical peaks at ~1002 cm^−1^ and ~1172 cm^−1^ in 1 M H_3_PO_4_ (10 wt%), which is assigned to the stretching vibration of P–O and P=O bond respectively. These two peaks gradually shift to the lower wavenumber along with the increase of concentration and finally shift to ~881 cm^−1^ and ~985 cm^−1^ in PPA^33^. The wide and strong absorption peak between 3200~3400 cm^−1^, attributed to the hydrogen bonding, is obvious in 1 M H_3_PO_4_ but totally disappears in PPA, indicating the absence of free water molecules in PPA. The similar structural transformation of the liquid structure was further confirmed by the Raman spectroscopy in Supplementary Fig. 1. A single peak attributed to the vibration of P=O bond splits into several peaks in PPA, indicating the complexity of the bond in PPA. No specific n value was given from reagent information and corresponding MSDS of PPA but an average n value could be inferred according to the P_2_O_5_ content. In fact, based on the investigation of previous literature^34–36^, PPA is more likely a mixture of homologous series with different degree of polymerization. The degree of polymerization can be described more precisely with P_2_O_5_ content other than a specific n value. The disappearance of water peak verifies the anhydrous characteristic of PPA, which is further confirmed by the result of coulometric Karl Fischer titration (H_2_O content is 426.3 ppm, 0.0426 wt%). Figure 1b displays the change of ^1^H chemical shift in the nuclear magnetic resonance (NMR) spectra. The ^1^H chemical shift gradually moves to the low-field direction with the decrease of water content, which can be attributed to the decrease of the electron cloud density in ^1^H nuclear due to inductive effect from neighboring P=O bond. All the three water-contained electrolytes show only ^1^H peak, indicating the ^1^H of hydroxyl and ^1^H of water are in constant dynamic exchange through hydrogen bonds. This exchange of H also set the structural basis of the rapid shuttle of protons inside the solvent-free PPA electrolyte.

Besides the structure, the dynamics of PPA were also investigated through the viscosity and ionic conductivity, as shown in Supplementary Figs. 2 and 1c. As expected, the viscosity decreases significantly at higher temperature, thus leading to higher ionic conductivity. The viscosity will decrease from 98.6 Pas at 25 °C to 0.37 Pas at 140 °C. Meanwhile, the ionic conductivity of PPA is only 0.45 mS cm^−1^ at room temperature, while it can be improved to 63.8 mS cm^−1^ at 200 °C, close to that of 1 M H_3_PO_4_ (Supplementary Fig. 3). Furthermore, the linear Arrhenius plot indicates ionic conductive mechanism is constant through the temperature range and the ionic conductivity is only limited by the high viscosity at low temperature, which is consistent with the single-ionic conductivity of PPA^37,38^. Differential scanning calorimetry (DSC) analysis was employed to test the boiling point of PPA. As shown in Fig. 1d, the boiling point of 1 M H_3_PO_4_ is only 105 °C, due to the existence of H_2_O. In sharp contrast, there is no endothermic or exothermic peak under 400 °C, confirming that PPA keeps stable at this wide temperature range (0–400 °C). We also marked the boiling points of common-used solvents in electrolytes for comparison. It can be seen clearly that none of the liquid solvents could survive beyond 250 °C. Moreover, the flammability test was conducted to verify the combustibility of PPA. As displayed in Supplementary Fig. 4, the glass fiber membrane infiltrated with PPA did not burn with combustion source. The DSC results and flammability test strongly demonstrate the superiority of PPA over other liquid electrolyte regarding the high-temperature operation and intrinsic safety. Considering PPA is deliquescent, contrast experiments were conducted to verify that the electrochemical device could keep PPA from absorbing water from the air (Supplementary Figs. 5 and 6). Even adding water to the PPA, the water layer remain above the PPA liquid layer, whose interior stay unchanged. The electrochemical stability window of these electrolytes was evaluated with linear scanning voltammetry (LSV) on titanium mesh electrodes (Fig. 1e and Supplementary Fig. 7). The overall stability window of H_3_PO_4_ aqueous solutions expands slightly as the concentration increases, while both oxygen and hydrogen evolution potentials are still limited by the thermodynamic stability of water. In comparison, the PPA exhibits a wider electrochemical window (>2.5 V). Owing to the absence of solvent that could be electrochemically oxidized, the anodic limit of PPA is even higher than 2 V (vs. Ag/AgCl), beyond any reported liquid electrolytes. Moreover, electrolyte stability voltage window was also measured with a 1 cm^2^ Pt electrode as working electrode under 60 °C at a scan rate of 0.1 mV s^−1^ to exclude the effect of the low conductivity of PPA and passivation of the Ti surface **(**Supplementary Fig. 8). The electrochemical window still expands obviously and the anodic limit of PPA is even higher than 2.5 V (vs. Ag/AgCl). It is worth noting that the reaction between lithium plate and PPA is rather slow, indicating that H^+^ in PPA is different from that in the normal aqueous solutions (Supplementary Fig. 9). It can be concluded that the activity of H^+^ in PPA is lower, which is consistent with the decrease of hydrogen evolution potential. Combining aforementioned characterizations, the solvent-free characteristics endow PPA with special properties, including high thermo stability, nonflammability, moderate ionic conductivities, and expanded electrochemical windows.

### Inhibiting MoO_3_ anode dissolution

**Fig. 2:**
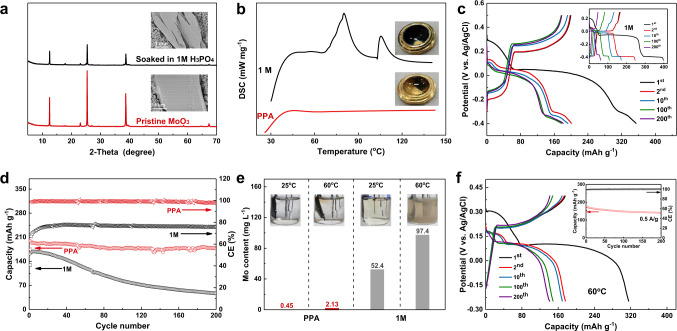
Characterization investigations and electrochemical performance of MoO_3_ anode. **a** XRD patterns of the pristine MoO_3_ electrode and MoO_3_ electrode soaked in 1 M H_3_PO_4_ for 5 days. **b** DSC for the mixture of MoO_3_ membrane and PPA (or 1 M H_3_PO_4_). **c**, **d** The comparison of voltage profiles and cycling performance of MoO_3_ cycled in the 1 M H_3_PO_4_ and PPA electrolytes at a current density of 0.2 A g^−1^ at 25 °C. **e** The concentrations of dissolved Mo in different electrolytes measured after 5 days cycling using ICP and corresponding digital images. **f** The electrochemical performance of MoO_3_ cycling at 60 °C with PPA electrolyte.

To verify the superiority of PPA, the model materials MoO_3_ was chosen as active working electrode in the proton batteries **(**Supplementary Figs. 10 and 11**)**. MoO_3_ has been widely used as anode for proton batteries in recent years^39–42^. However, it is well known that metallic oxide could dissolve in aqueous acid solutions, which was verified by the weakened peak and damaged surface of MoO_3_ electrode soaked in 1 M H_3_PO_4_ for 5 days (Fig. 2a**)**. The dissolution of transition metal from electrode materials after exposure in different electrolytes was quantified by inductively coupled plasma (ICP) spectroscopy (Supplementary Fig. 12). The Mo contents are 110.5 and 119.9 mg/L when soaked in 1 M H_3_PO_4_ for 5 days at 25 °C and 60 °C, respectively, which confirms the severe dissolution of MoO_3_ in 1 M H_3_PO_4_. By contrast, only very small amount of Mo was detected in the PPA electrolyte whether under room temperature or elevated temperature. The comparison could confirm the superiority of PPA in preventing dissolution of metal oxide. Furthermore, differential scanning calorimetry (DSC) was conducted to evaluate the reaction between MoO_3_ and the electrolyte. As shown in Fig. 2b, the wide endothermic peak below 100 °C when MoO_3_ and 1 M H_3_PO_4_ were co-heated indicates that MoO_3_ suffers serious dissolution in aqueous acid solution. Nevertheless, there is no obvious peak when MoO_3_ and PPA were co-heated, suggesting that PPA can suppress the MoO_3_ dissolution.

The electrochemical behaviors of MoO_3_ in 1 M H_3_PO_4_ and PPA electrolytes were evaluated using three-electrode cells. Figure 2c presents the galvanostatic discharge-charge profiles of MoO_3_ in PPA at 0.2 A g^−1^. Consistent with previous reports^39,41^, MoO_3_ delivered a partially reversible three-step proton insertion during the first discharging, corresponding to a high capacity of 360.9 mAh g^−1^. During subsequent cycles, a reversible capacity of 198.9 mAh g^−1^ was achieved with a high capacity retention of 89.4% over 200 cycles and a high columbic efficiency (CE) over 99% (Fig. 2d).

In sharp comparison, although a similar three-step behavior and a lower polarization of voltage platforms was observed in 1 M H_3_PO_4_ (inset of Fig. 2c), the corresponding CE was below 80% during the entire cycles and much lower than that in PPA, which is likely due to the severe hydrogen evolution in 1 M H_3_PO_4_ electrolyte. Besides, the MoO_3_ electrode suffered from continuous capacity decay over cycling and only 28.7% of its initial reversible capacity remained after 200 cycles (Fig. 2d). Increasing the discharging cut-off voltage did not benefit the cycling performance (Supplementary Fig. 13), indicating that the dissolution of MoO_3_, other than the electrolyte decomposition, should be blamed for the capacity decay in the 1 M H_3_PO_4_. To get a more rigorous comparison of the performance of MoO_3_ in different electrolytes, the cycling performance of MoO_3_ in the PPA electrolyte using a much wider voltage range (−0.4–0.5 V) was compared with that using a narrow voltage (−0.2–0.3 V) in the 1 M H_3_PO_4_ electrolyte. As shown in Supplementary Fig. 14, even using a much wider voltage range, the cycling stability and Coulombic efficiency of MoO_3_ in the PPA electrolyte is better than that in the 1 M H_3_PO_4_.

To further quantify the dissolution, ICP was utilized to analysis the electrolytes after cycling for 5 days. As shown in Fig. 2e, the concentrations of the dissolved Mo in the PPA electrolytes cycled at 25 °C and 60 °C are only 0.45 and 2.13 mg/L, while the Mo concentration in 1 M H_3_PO_4_ cycling at 25 °C and 60 °C are as much as 52.4 and 97.4 mg/L, respectively, which is consistent with the visual digital images. Combining the above results, it can be concluded that excluding the water solvent stabilizes the MoO_3_ electrode and allows the reversible insertion/extraction of proton, otherwise the dissolution of MoO_3_ would happen in the water contained 1 M H_3_PO_4_ electrolyte and deteriorate in elevated temperatures.

The performance of the MoO_3_ half cell was thus examined at the elevated temperature of 60 °C using various rates (Supplementary Fig. 15). The lower cut-off voltage was set as −0.2 V to avoid the hydrogen evolution. The charging capacities of 212, 191, 163, 141, and 123 mAh g^−1^ were delivered at rates of 0.1, 0.2, 0.5, 1.0, and 1.5 A g^−1^, respectively. Figure 2f exhibits the performance at 0.5 A g^−1^ and a high initial charging capacity of 175 mAh g^−1^ was achieved, with a smaller polarization than that of 25 °C. The capacity maintained as high as 142 mAh g^−1^ after 200 cycles with a high CE above 99.5%, much better than that in 1 M H_3_PO_4_ (Supplementary Fig. 16) and further confirming the superiority of water-free PPA electrolyte.

### Inhibiting LVPF cathode dissolution

**Fig. 3:**
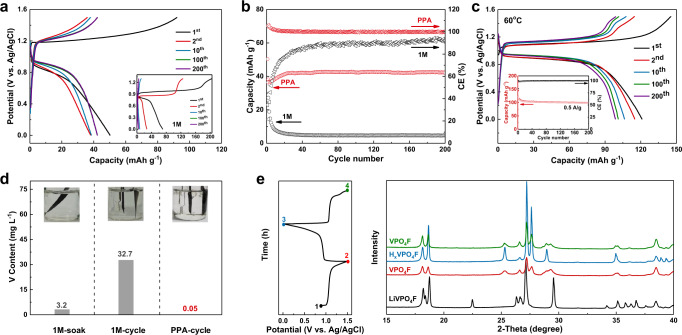
Characterization investigations and electrochemical performance of LVPF cathode. **a**, **b** The comparison of voltage profiles and cycling performance of LVPF that cycled in the 1 M H_3_PO_4_ and PPA electrolytes at a current density of 0.2 A g^−1^ at 25 °C. **c** The electrochemical performance of LVPF cycled at a current density of 0.5 A g^−1^ at 60 °C in PPA electrolyte. **d** The concentrations of dissolved V in different electrolytes measured after 5 days using ICP and corresponding digital images (Note that the instrument detection limit is 0.05 mg/L). **e** XRD patterns of the pristine LiVPO_4_F (black), full charged VPO_4_F (red), full discharged H_x_VPO_4_F (blue), and re-charged VPO_4_F (green).

To couple with MoO_3_, a high voltage proton cathode, which should start from charging, needs to be chosen. To date, most reported materials in proton battery act as anodes with low potentials and need to be discharged firstly^43–48^. Herein, we propose and demonstrate LiVPO_4_F (LVPF) as the cathode for the proton batteries^32,49–52^. The X-ray diffraction (XRD) pattern and scanning electron microscopy (SEM) image of LVPF are given in Supplementary Figs. 17 and 18. The electrochemical performance of LVPF was firstly evaluated in PPA and 1 M H_3_PO_4_ using three-electrode tests at 0.2 A g^−1^, as shown in Fig. 3a, b, in 1 M H_3_PO_4_, LVPF could deliver a capacity of 204 mAh g^−1^ during the first charging process, corresponding to the extraction of lithium. Noting that the theoretical delithiation capacity of LVPF is 156 mAh g^−1^, the excess charging capacity is possibly derived from oxidative decomposition of H_2_O. However, the discharging capacity associated with the insertion of proton was only 70 mAh g^−1^, followed by a rapid decrease to 8 mAh g^−1^ after only ten cycles. In direct contrast, the fading problem has been essentially circumvented by using PPA electrolyte, where negligible capacity loss was observed over 1000 cycles (Supplementary Fig. 19). The relatively low capacity should be ascribed to the high viscosity and relatively low conductivity of PPA at 25 °C. Since the PPA electrolyte contains both Li^+^ and H^+^ after the initial lithium extraction, it is necessary to clarify whether proton insertion dominated the following process. Therefore, X-ray photoelectron spectroscopy (XPS) was carried out to investigate the Li existence of LVPF electrodes at various states and the result is shown in Supplementary Fig. 20. It is obvious that the Li *1s* peak disappeared when LVPF was fully charged, indicating the completely delithiation and the formation of VPO_4_F after the first charging process. During subsequent cycles, no Li peak was observed any more, indicating that the capacity totally arises from the proton insertion. In addition, the charged LVPF electrode was taken out from PPA, washed and then cycled in a new PPA electrolyte to exclude contribution of Li^+^ originating from delithiation. The voltage profiles and capacity kept identical to the one without replacing PPA (Supplementary Fig. 21). As a result, it can be convinced that only H^+^ involved in the reaction of LVPF electrode in PPA.

Therefore, the electrochemical performance of LVPF in PPA at elevated temperature (60 °C) was investigated. Resulting from the smaller viscosity, LVPF shows smaller polarization at 60 °C and has a reversible capacity of 121 mAh g^−1^ in the first cycle at the rate of 0.5 A g^−1^ (Fig. 3c). owing to the insolubility and structural stability during cycling, a high capacity retention of 82% is still attainable over 200 cycles at a moderate current density of 0.5 A g^−1^ (inset of Fig. 3c).

The degradation of LVPF in 1 M H_3_PO_4_ was also investigated using ICP. As shown in Fig. 3d, Soaking the LVPF electrode in 1 M H_3_PO_4_ for 5 days did not cause color change, and only 3.2 mg/L V was detected. After cycling LVPF in 1 M H_3_PO_4_ for 5 days, the electrolyte turned blue, which is the typical color of V(IV), and the concentration of dissolved V was as high as 32.7 mg/L, confirming that the charged LVPF experienced the serious dissolution and structural collapse in the water-contained 1 M H_3_PO_4_ and thus leading to poor cycling performance. In contrast, the dissolved V were not detectable in the cycled PPA, indicating the stabilization of LVPF lattice. As evidenced by the shift of XRD pattern (Fig. 3e), after the initial charging process, LVPF totally transformed into VPO_4_F^52^, which is also verified by the STEM EDS-elemental mapping images of the delithiated LVPF (Supplementary Fig. 22). The characteristic peak for LVPF at 22.5° disappeared after the initial charging and did not appear in the subsequent discharging process, confirming that the Li^+^ did not insert into delithiated VPO_4_F, which is mainly because the concentration of proton is order of magnitudes higher than that of Li^+^ derived from delithiation. In the subsequent cycles, no obvious changes were observed in the XRD patterns, confirming that the reversible insertion of H^+^ (from PPA) has little effect on the VPO_4_F lattice frame. In addition, XRD patterns of electrode after different cycles have been compared in Supplementary Fig. 23. The XRD patterns of electrode after 10 cycles and 50 cycles are almost identical to that cycled for 1 cycle, and no characteristic peak of LVPF appears any more during the cycling, which confirms the highly reversible transformation between VPO_4_F and H_x_VPO_4_F. Owing to the nearly zero-strain insertion mechanism, structural collapse and further dissolution could be avoided, by replacing the H_3_O^+^ carrier ions with non-solvent protons (H^+^) in PPA (Supplementary Fig. 24).

### DFT simulations

**Fig. 4:**
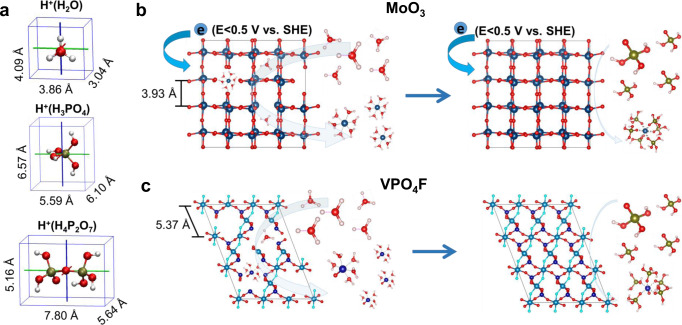
Schematic of DFT simulations. **a** The ionic size of various solvated proton structures. The schematic comparisons of the proton intercalation into **b** the reduced MoO_3_ and **c** VPO_4_F lattice framework in the diluted 1 M H_3_PO_4_ (left) and PPA (right) electrolytes.

The dissolution of the electrodes in the diluted 1 M H_3_PO_4_ and PPA electrolytes were also investigated using the DFT calculations. The ionic size of H_3_O^+^ in 1 M H_3_PO_4_ electrolyte (3.04 Å) is much smaller than that of H^+^(H_3_PO_4_) (5.59 Å) and H^+^(H_4_P_2_O_7_) (5.16 Å) clusters (Fig. 4a). The small size of the solvated proton structure enables a co-intercalation of the water molecules since the tunnel size for MoO_3_ and VPO_4_F are measured to be 3.93 and 5.37 Å, respectively. The intercalated water can easily solvate with the Mo and V atom with large solvation energy (Supplementary Fig. 25). The dissolution process takes place not only at the surface but also inside the particles by the co-intercalated water molecules (Fig. 4b, c). However, for the proton cluster in the PPA electrolyte, the intercalation has been blocked due to the steric hindrance. The protons have to desolvate before insertion, accelerating the migration. The solvation energies for Mo^3+^ and VO^2+^ with H_3_PO_4_ molecules indicate that MoO_3_ and VPO_4_F could be easily dissolved into PPA. However, the dissolution is limited at the interface of the electrode and the PPA electrolyte, confirming the advantage of solvent-free PPA electrolyte in suppressing the dissolution of the electrode. Moreover, the electrochemical dissolution of the MoO_3_ and VPO_4_F was evaluated by using the pourbaix diagram (Supplementary Fig. 26). MoO_3_ is electrochemical stable when the voltage is higher than 0.5 V versus SHE in the solution with PH < 2. The MoO_3_ would be reduced to Mo^3+^ when the voltage lower than 0.5 V. VPO_4_F is unstable in the aqueous solution for all the potentials and PH values according to the pourbaix diagram, which is consist with the experimental result that VPO_4_F suffers severe dissolution in the 1 M H_3_PO_4_ electrolyte^32^.

### Electrochemical performance of the proton full battery

**Fig. 5:**
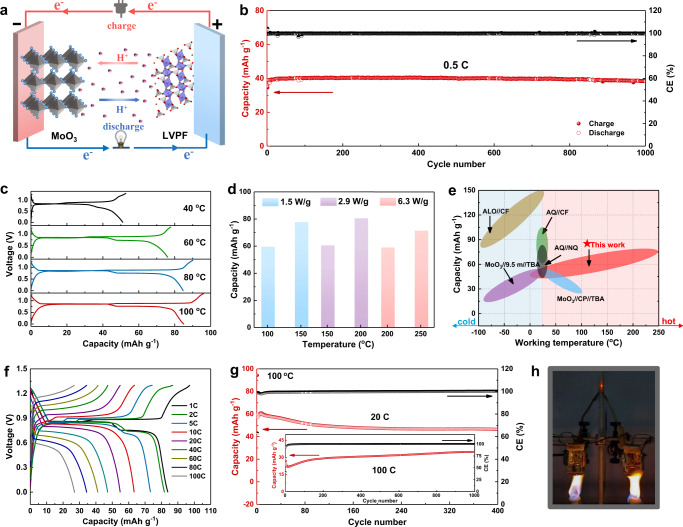
Electrochemical performance of the MoO_3_||LVPF full cell. **a** Schematic of the battery. **b** Cycling performance at a rate of 0.5 C at room temperature. **c** Voltage profiles at different temperatures at a rate of 0.5 C. **d** Reversible specific capacity in different temperatures. **e** Comparison of the working temperature and capacity for this work and previously reported proton batteries. **f** The rate performance of the cell from 1 C to 100 C at 100 °C. **g** Cycling performance of the cell at 100 °C. **h** Optical images of an LED powered by two beaker batteries in series under baking.

To demonstrate the feasibility of PPA electrolyte in a wide operating-temperature range, a rocking-chair full proton cell was assembled using MoO_3_ anode and LVPF cathode with a N/P ratio of 2/3, as schematically shown by Fig. 5a. The testing parameters were calculated based on the theoretical capacity of full cell (1 C = 93 mA g^−1^) using the mass of both cathode and anode electrodes. Firstly, the room-temperature performance was evaluated and shown in Supplementary Figs. 27 and 5b. From the galvanostatic discharge-charge profiles in Supplementary Fig. 27, three platforms were observed. The cell delivered a capacity of 40 mAh g^−1^ and cycled stably at 0.5 C with negligible capacity fading over 1000 cycles, suggesting prominent cycling stability. To demonstrate the low-temperature (T) capability, the cell was discharged at 25 °C, 10 °C, and 0 °C respectively after charging to 1.3 V with a charge capacity of 78 mAh g^−1^ at 60 °C. The discharge capacities were 60, 49, and 46 mAh g^−1^ at 25 °C, 10 °C and 0 °C, respectively (Supplementary Fig. 28). To investigate the charge transfer of the interface, we conducted electrochemical impedance spectroscopy (EIS) measurements on a MoO_3_/LiVPO_4_F battery and the result is shown in Supplementary Fig. 29. The diameters of the semicircles in the Nyquist complex plots mark the charge-transfer resistance (R_ct_), which increase dramatically as the temperature decreases whether at charged or discharged states. The results suggest that slower proton conduction across the electrode/electrolyte interface and lower ionic conductivity of PPA electrolyte lead to the low discharge capacity at low temperature together. In addition, the galvanostatic discharge-charge test has also been further examined to confirm the operation of the battery at low temperature and the result is shown in Supplementary Fig. 30. Considering the rather low ionic conductivity and increased polarization, the charge cut-off voltage was increased to 1.5 V and the test rate was set as 0.1 C. The reversible capacities at 0 °C and −10 °C were 26.2 and 10.1 mAh g^−1^, respectively. It should be noted that the ionic conductivity of PPA is 0.45 mS cm^−1^ at 25 °C and the viscosity would be higher at lower temperature, where charge carrier diffusion will be rather sluggish. The viability of the cell at low-T fully demonstrates the acceptable proton transport in PPA, even with a high viscosity.

More importantly, this proton battery exhibits unprecedented high-temperature performance owing to the high boiling point and thermostability of PPA. Figure 5c presents the charge/discharge curves of the MoO_3_-LVPF full cell at 0.5 C at various high temperatures. The discharge capacities at 40, 60, 80, and 100 °C were 51, 77, 84, and 85 mAh g^−1^, respectively. It is worth noting that the capacity of 84 mAh g^−1^ at 80 °C is twice higher than that at 25 °C (40 mAh g^−1^) and close to the theoretical capacity (93 mAh g^−1^). The enhanced capacity could be attributed to the reduced viscosity and decreased cell resistance at higher temperature. Surprisingly, this proton full cell delivered a high reversible capacity of 80 mAh g^−1^ at 200 °C with a power density of 2.9 W/g and 71 mAh g^−1^ at 250 °C with a power density of 6.3 W/g (Fig. 5d**)**. Comparison of this work and previously reported proton batteries in Fig. 5e and Supplementary Table 1 clearly shows the ultra-wide temperature range and relatively high capacity and energy density of this proton battery compared with other reported proton batteries, which is especially suitable for application at high temperature^40,53–56^.

After confirming the high-T capability of the full cell, 100 °C was selected as a typical high-T condition to investigate the detailed electrochemical performance. The cell exhibited a reversible capacity of 84 mAh g^−1^ based on the active mass of both electrodes at 1 C rate, close to the theoretical value. The excellent rate capability with 32% capacity retention upon raising the rate from 1 C to 100 C is demonstrated in Fig. 5f, where the proton battery discharged within 10.3 s with a maximum power density of 4975 W kg^−1^ at 100 C (see Supplementary Note 1 for detailed calculations). In addition, as the rate decreased, the platform at 1.25 V, which could be assigned to the low-potential plateau of MoO_3_, gradually appeared in the full cell due to the reduced cell polarization. Besides the high rate performance, the cell could cycle stably at 20 C and displayed good cycling stability with no capacity fading at 100 °C and 100 C over 1000 cycles (Fig. 5g). Furthermore, two beaker cells integrated in series were employed to light up light emitting diode (LED) under the flame of alcohol lamps (~500 °C). The continuous red light (Fig. 5h) verified the intrinsic thermostability and safety of the full cell under high temperature conditions, which is impossible for any previous liquid electrolyte batteries. Aforementioned results successfully demonstrate that the solvent-free PPA electrolyte enables MoO_3_||LVPF proton cell to operate well within an ultra-wide temperature range of 0 °C to 250 °C, making it especially suitable for the applications at high temperature requiring high power density and high reliability, such as the fire rescue/inspection robots and space exploration.

## Discussion

In summary, polyphosphoric acid (PPA) was demonstrated as a solvent-free protic liquid electrolyte which reconciles the merits of conventional liquid and solid electrolytes while resolving each of their inferiorities. Benefiting from the solvent-free feature, PPA possesses nonflammability, wide electrochemical stability window (>2.5 V), low volatility and wide working temperature range (>400 °C). Meanwhile, combining the experimental results and DFT simulations, it was shown that the solvent-free proton intercalation in PPA could suppress the electrodes degradation compared with diluted 1 M H_3_PO_4_. As a result, a rocking-chair cell with MoO_3_ anode and LVPF cathode was demonstrated. The battery not only delivers power in unprecedented ultra-wide temperature range of 0–250 °C, but also operates well at ultra-high rate (1–100 C) and could even light up LED under the flame of alcohol lamps (~500 °C), which is superior than current liquid batteries. The demonstration of solvent-free electrolyte provides a viable route for the stable and safe batteries working under harsh conditions, especially for the use in various high-T temperature applications requiring high power density and high security, such as rescue/inspection robots and space exploration. The approach of excluding solvents to evade its inherent defects has the potential to open up a route towards the electrolyte design.

## Methods

### Materials preparation

LiVPO_4_F (LVPF) was purchased from Advanced lithium Electrochemistry (Cayman) Co. Ltd. MoO_3_ materials and polyphosphoric acid (PPA, H_n+2_P_n_O_3n+1_, ≥85% P_2_O_5_) were purchased from Aladdin Corporation. The concentration of PPA used in our study can be described as ≥85% P_2_O_5_, which represents an average degree of polymerization. 85 wt% H_3_PO_4_ was diluted with water to prepare 1 M H_3_PO_4_ and 50 wt% H_3_PO_4_.

### Materials characterizations

X-ray diffraction (XRD) patterns were obtained by Bruker D8 Endeavor X-ray diffractometer, employing Cu-Kα radiation (40 KV, 40 mA), to analyze the structure of MoO_3_ and LVPF. The morphologies of MoO_3_ and LVPF were characterized by scanning electron microscope (SEM, FEG-S4800). ICP measurement was performed using a vario EL Elemental Analyzer (Analysemsysteme GmbH, Germany) to analyze the elemental content in different electrolytes after 5 days cycling or soaking. Attenuated total reflection Fourier transform infrared (ATR-FTIR) spectroscopy was conducted on a Nicolet 6700 FTIR spectrometer instrument to examine the structure of electrolytes. Raman spectra were obtained on confocal microscope (Horiba LabRAM HR). The coulometric Karl Fischer titration was used to determine the water content in PPA with a 756 KF coulometer (Metrohm). The ^1^H nuclear magnetic resonance (NMR) spectra was conducted with a 500 MHz Bruker Avance III spectrometer. The shear viscosity was obtained using a Rotary Rheometer (HAAKE MARS III, Thermo Fisher). The differential scanning calorimetry (DSC) tests were acquired with a DSC 200 F3 Maia (NETZSCH, Germany) at a heating rate of 5 °C/min and a cooling rate of 10 °C/min under N_2_ atmosphere. X-ray photoelectron spectroscopy (XPS) was carried out to investigate the elemental existence and valent state of electrodes at various charge and discharge states, with a Thermo Scientific K-Alpha^+^ device.

### Preparation of electrodes and battery assembly

The MoO_3_ and LVPF electrodes were fabricated by compressing active materials, acetylene black and polytetrafluoroethylene (PTFE) at a weight ratio of 7: 2: 1. Activated carbon (AC) membrane was prepared by mixing activated carbon with acetylene black and PTFE in a mass ratio of 8:1:1. The active materials membrane were obtained by rolling the mixture into a membrane and going through drying process at 120 °C overnight. The electrode used for tests were prepared by pressing the required materials membrane on titanium mesh current collector. The metal electrode was prepared with a titanium mesh or platinum plate (1 cm*1 cm). The three-electrode system consists of MoO_3_/LVPF electrode (about 2.5 mg active material) or metal electrode as working, excess active carbon as the counter electrode and Ag/AgCl (saturated KCl) as the reference electrode (E = 0.199 V versus standard hydrogen electrode) whether in 1 M phosphoric acid or in PPA electrolytes. The volume of electrolyte used in tests (half-cell operation and electrode soaking) is 12 mL. The full cell was assembled in CR 2016-type coin cells with Glass fiber as separator and the amount of electrolyte was 100 uL (206 mg). The electrode was prepared by pressing the elelctrode materials membrane on titanium mesh current collector with 10 mm diameter. The areal mass loading of the anode and cathode were 1.5 mg/cm^2^ and 2.25 mg/cm^2^, respectively.

### Electrochemical measurements

The electrochemical performances of MoO_3_ and LVPF were investigated, respectively, by a typical three-electrode system. Cyclic voltammetry and linear sweep voltammetry were carried out using AUTOLAB electrochemical work station (PGSTAT 302N). The galvanostatic charge/discharge performance and rate performance were collected by using Hukuto Denko battery charge/discharge system HJ series (Hukuto Denku, Japan), Land BT2000 battery test system (Wuhan), and Neware battery test system (CT-4008-5V5mA-164, Shenzhen, China). The ion conductivities of electrolytes were calculated by electrochemical impedance spectroscopy (EIS) results, which was operated with two parallel Ti-plate (1 cm * 1 cm) as electrodes. The measuring frequency range was kept between 0.1 Hz and 100 KHz. The low/high-temperature performance of the cell was tested in a freezer or oven. Before testing, the cell was kept under a specific temperature for 1 h to reach the equilibrium of cell temperature and pre-set temperature.

### Computational details

The quantum chemistry calculations were performed with Gaussian 09 software. Except with explicitly noted, Geometry optimizations and energy calculations were performed using the B3LYP/6-311G (*d*, *p*). For Mo element of Mo contained clusters, a small-core quasi-relativistic effective core potential (ECPs)^1^. and the cc-pVTZ valence triple zeta basis set^2^ were employed. The sizes of the solvated protons measured with the Multiwfn software^3^. The pourbaix diagrams were obtained from the Materials Project.

## Supplementary information

Supplementary Information

## Data Availability

The data that support the plots within this paper and other findings of this study are available from the corresponding author upon reasonable request.
