# Supplementary material for: Solvent-free protic liquid enabling batteries operation at an ultra-wide temperature range
Source: Nat Commun. 2022 Oct 13;13:6064. doi: 10.1038/s41467-022-33612-2 (PMC9561716; doi:10.1038/s41467-022-33612-2)
Supplement: Supplementary file 1 — Supplementary Information [file 41467_2022_33612_MOESM1_ESM.pdf]

Supplementary Information

Solvent-free protic liquid enabling batteries operation at an ultra-wide temperature range

Liao, *et al.*

Supplementary Figures

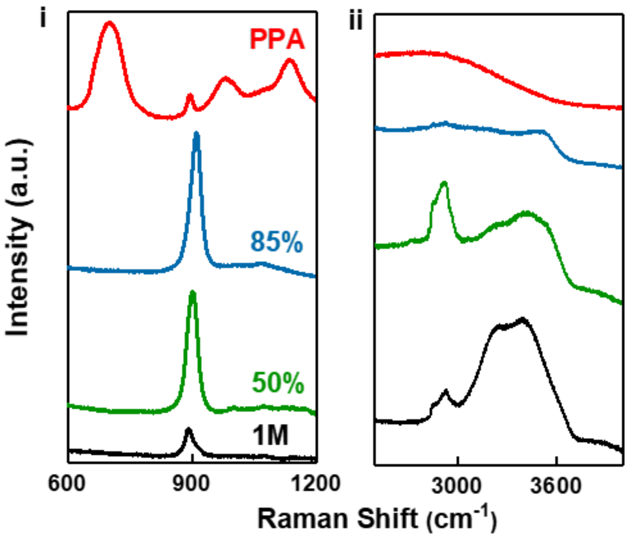

Supplementary Figure 1 | The Raman spectroscopy of 1 M H_3PO_4, 50 wt% H_3PO_4, 85 wt% H_3PO_4, and PPA.

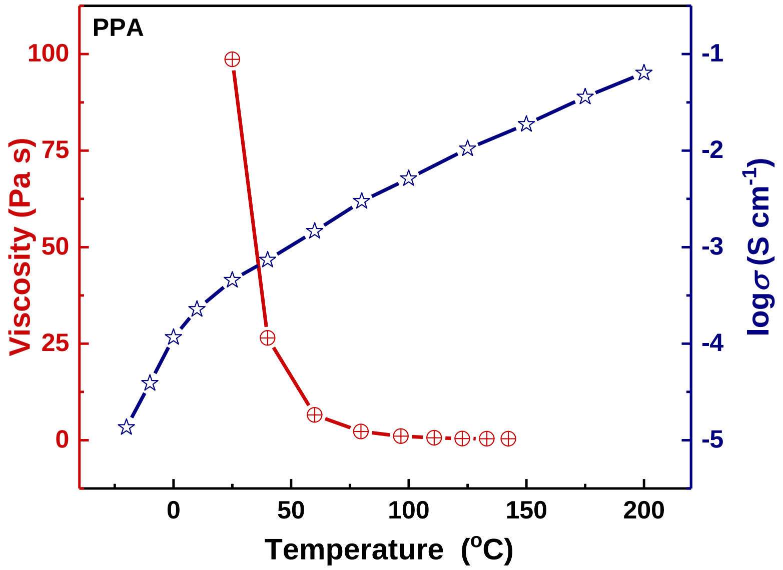

Supplementary Figure 2 | The viscosity and ionic conductivity of PPA electrolyte.

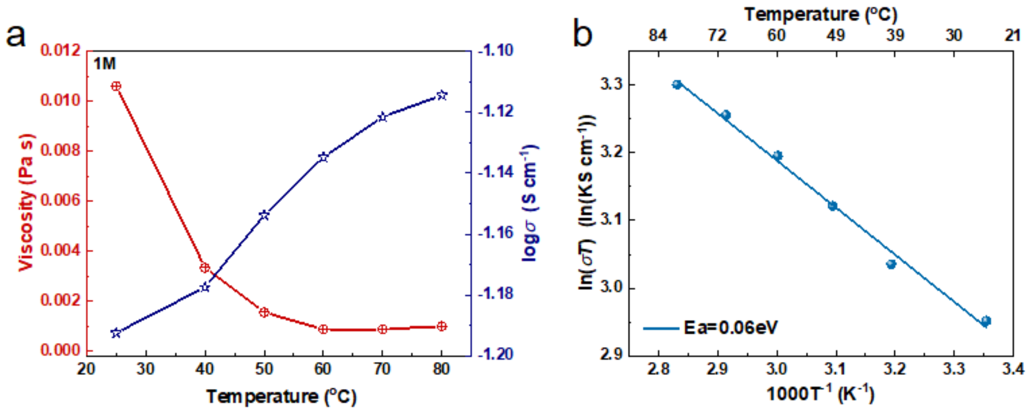

Supplementary Figure 3 | The viscosity, ionic conductivity and corresponding Arrhenius plot of 1 M H_3PO_4. **a**, The viscosity and ionic conductivity of 1 M H_3PO_4. **b**, The electric conductance activation energy of 1 M H_3PO_4.

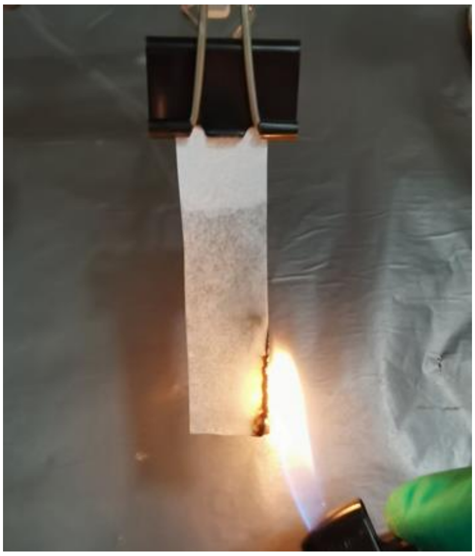

Supplementary Figure 4 | Flammability test of PPA electrolyte.

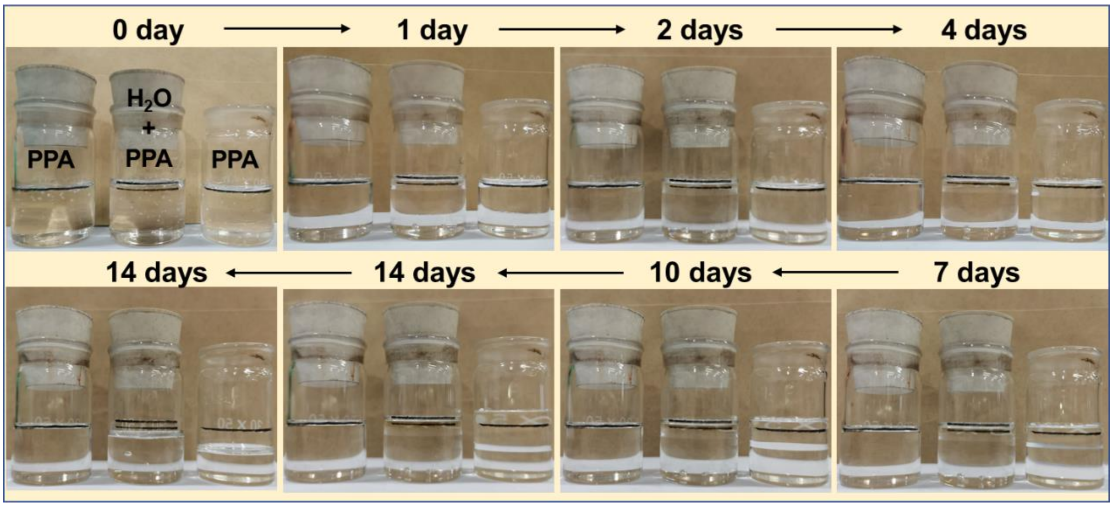

Supplementary Figure 5 | The optical photographs of three beakers containing PPA which are set in the air with a relative humidity of approximately 40%. (Both of the left beaker and the right beaker contain only PPA, but the left beaker is tight with a rubber stopper. The middle rubber is also tight with a rubber stopper with a 2 mm water layer added above the PPA. Three beakers contain the same amount of PPA in the very beginning.)

Supplementary Note: **Figure S5** shows the different degree of water absorption in different conditions. The liquid level of the right beaker goes up but the left beaker has no change after two weeks, suggesting the rubber stopper could inhibit the water in the air be absorbed in PPA. The water layer increased and PPA layer decreased in the middle beaker but the total height remains the same, indicating that water will spontaneously mix with the PPA and the rubber plug can prevent additional water absorption. After 14 days, we can easily pour the top water because the PPA and water are always in different layers, which illustrates that there is no change inside the PPA even if there is a water layer above the PPA layer. As a result, we can use such a beaker with rubber plug to do electrochemical tests.

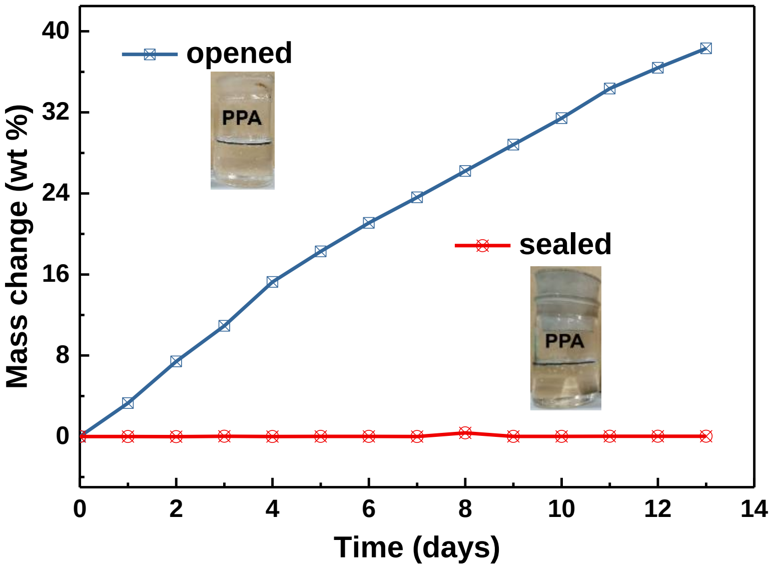

Supplementary Figure 6 | The quantitative weight gain/loss characterization data of the sealed beaker containing PPA used for electrochemical tests and a completely opened beaker containing PPA.

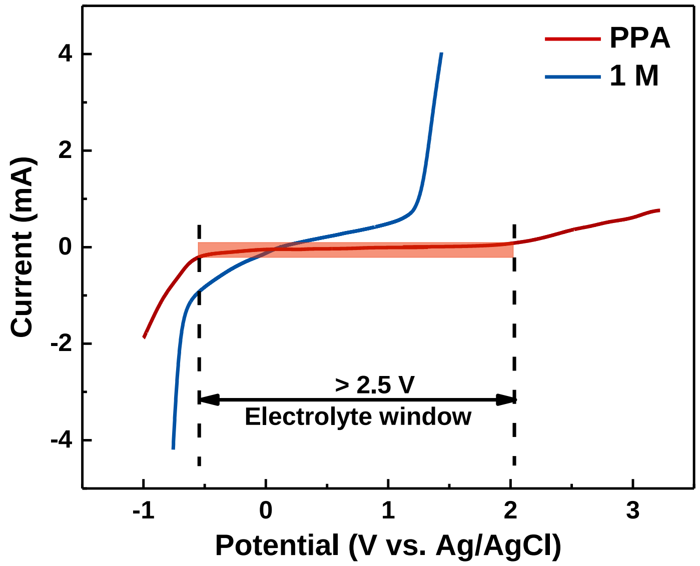

Supplementary Figure 7 | Linear sweep voltammetry (LSV) profiles of the of 1 M H_3PO_4 and PPA electrolytes with a scan rate of 1 mV s^{-1} with Ti mesh under 25 ºC.

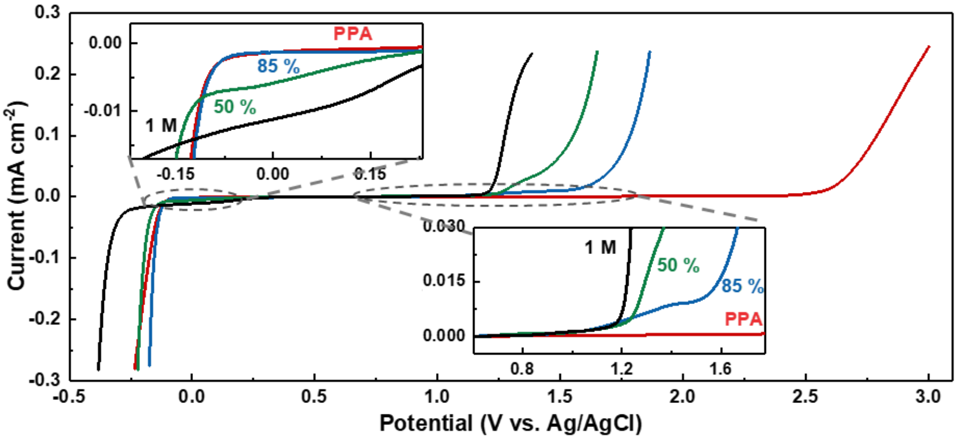

Supplementary Figure 8 | Electrochemical stability window of 1 M H_3PO_4, 50 wt% H_3PO_4, 85 wt% H_3PO_4 and PPA electrolytes measured with LSV on Platinum electrode at a scan rate of 0.1 mV s^{-1} at 60 ºC.

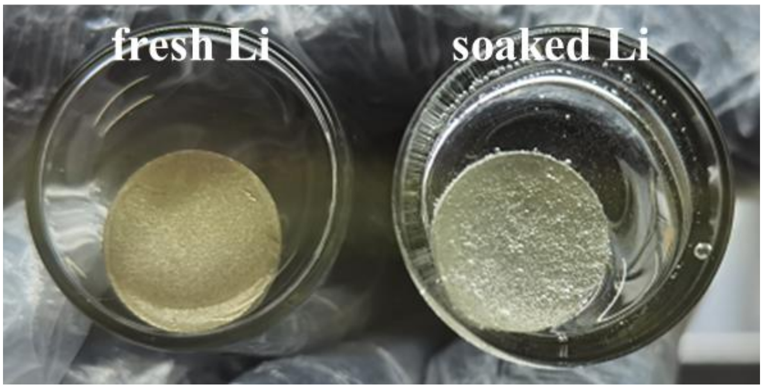

Supplementary Figure 9 | The digital images of fresh lithium plate (left) and lithium plate soaked in PPA for 48 hours (right).

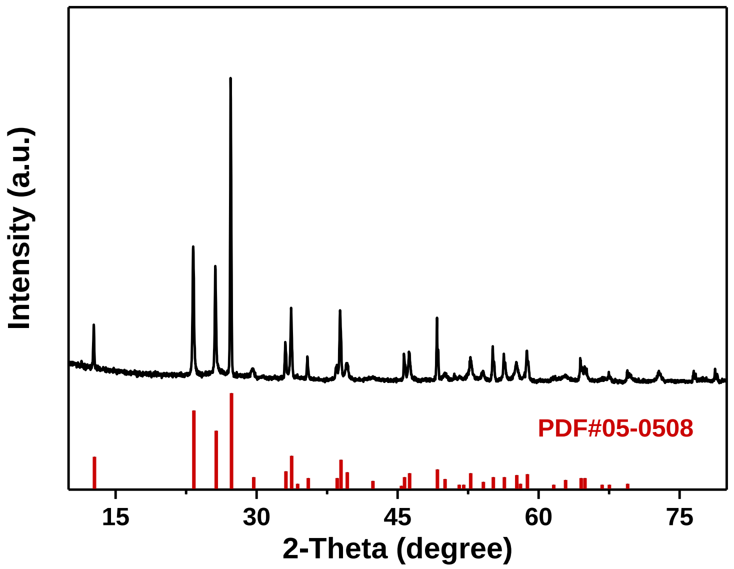

Supplementary Figure 10 | The XRD pattern of MoO_3.

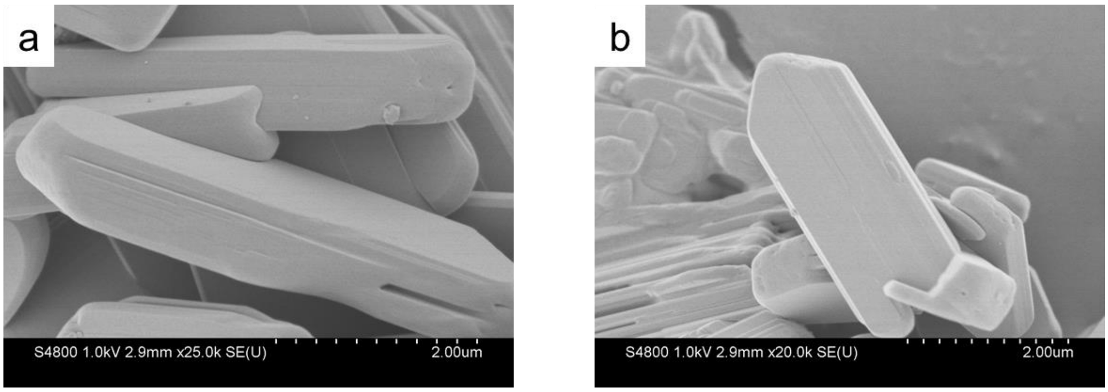

Supplementary Figure 11 | The SEM images of MoO_3.

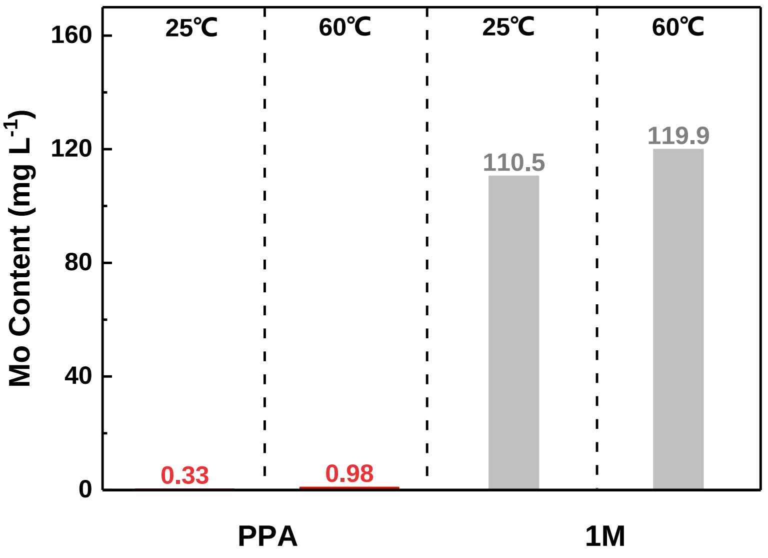

Supplementary Figure 12 | The concentrations of dissolved Mo after five days of material exposure in electrolytes at 25 ºC and 60 ºC.

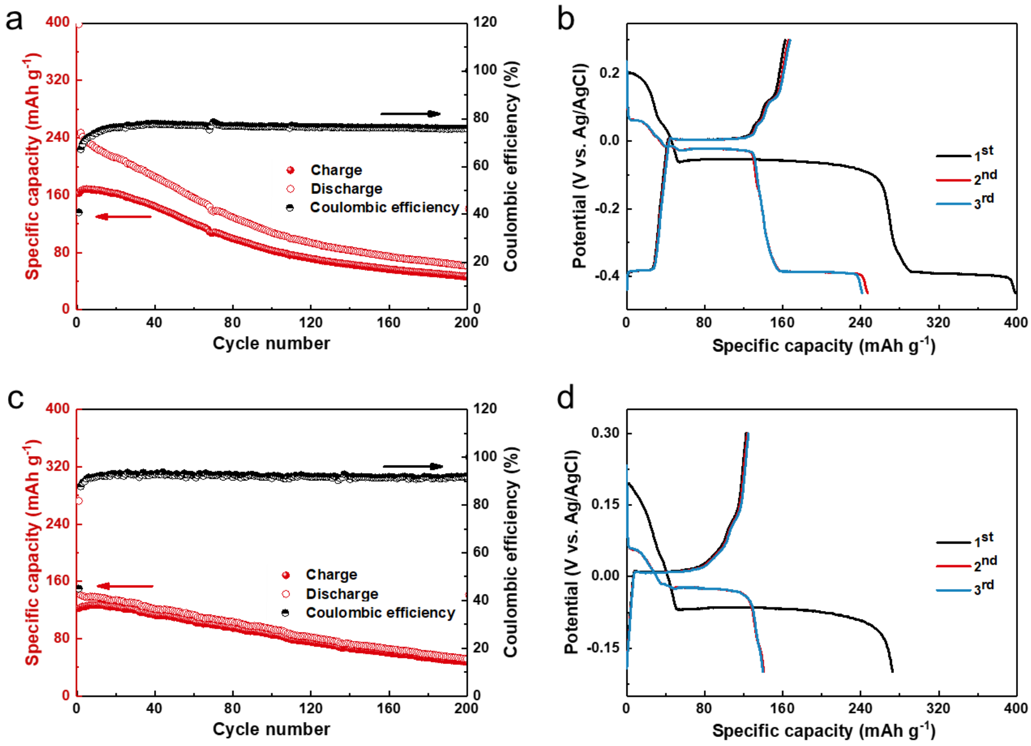

Supplementary Figure 13 | The electrochemical performance of MoO_3 in 1 M H_3PO_4 with different voltage range. The cycling performance (**a**) and voltage profile (**b**) of a MoO_3 half-cell in 1 M H_3PO_4 at 25°C with a rate of 0.2 A/g (-0.45-0.3 V). The cycling performance (**c**) and voltage profile (**d**) of a MoO_3 half-cell in 1 M H_3PO_4 at 25°C with a rate of 0.2 A/g (-0.2-0.3 V).

Supplementary Note: **Figure S13a and b** show the complete galvanostatic discharge-charge results containing three voltage platforms. It can be seen that a low Coulombic efficiency of approximately 76% maintains over 200 cycles. We wonder this phenomenon is related to the hydrogen evolution or not, so a comparative experiment **(Figure S13c and d)** is conducted where the voltage range is -0.2-0.3 V, which may suffer less hydrogen evolution. As expected, a Coulombic efficiency of approximately 92% is obtained, which is higher than the former one, but capacity fading is still severe. Therefore, it can be concluded that hydrogen will generate in the discharge process of MoO_3 in 1 M H_3PO_4, resulting in low Coulombic efficiency.

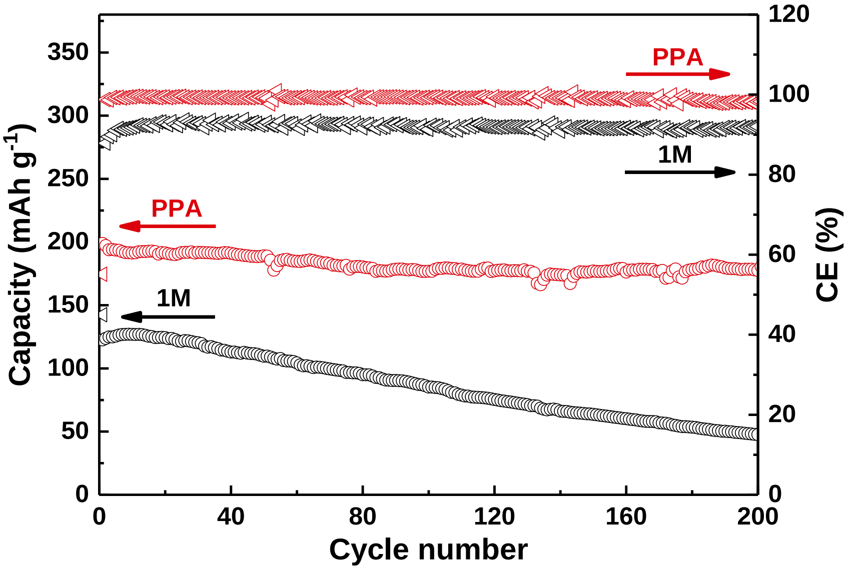

Supplementary Figure 14 | The comparison of cycling performance of MoO_3 cycled in the 1 M H_3PO_4 (-0.2-0.3 V) and PPA (-0.4-0.5 V) electrolytes at a current density of 0.2 A g^{-1} at 25 °C.

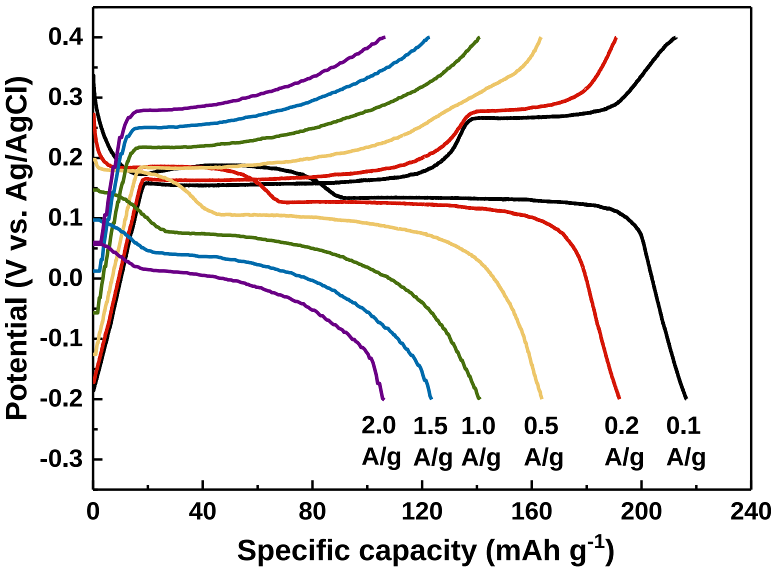

Supplementary Figure 15 | The rate performance of the MoO_3 half-cell in PPA electrolyte at 60°C.

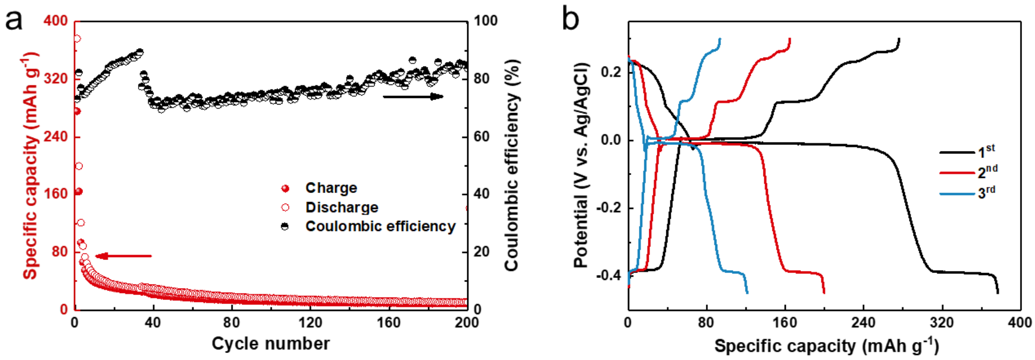

Supplementary Figure 16 | The electrochemical performance of MoO_3 in the 1 M H_3PO_4 electrolyte at 60 °C. The cycling performance (**a**) and voltage profiles (**b**) of MoO_3 that cycled in the 1 M H_3PO_4 electrolyte at a current density of 0.2 A g^{-1} at 60 °C.

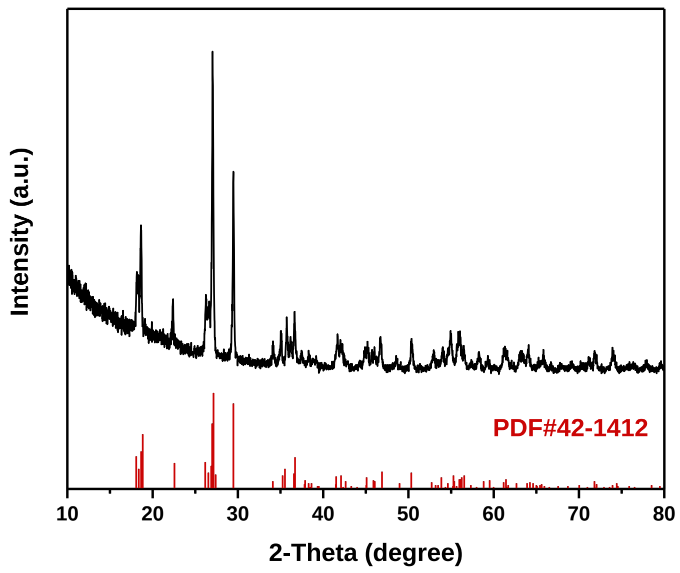

Supplementary Figure 17 | The XRD pattern of $LiVPO_4F$.

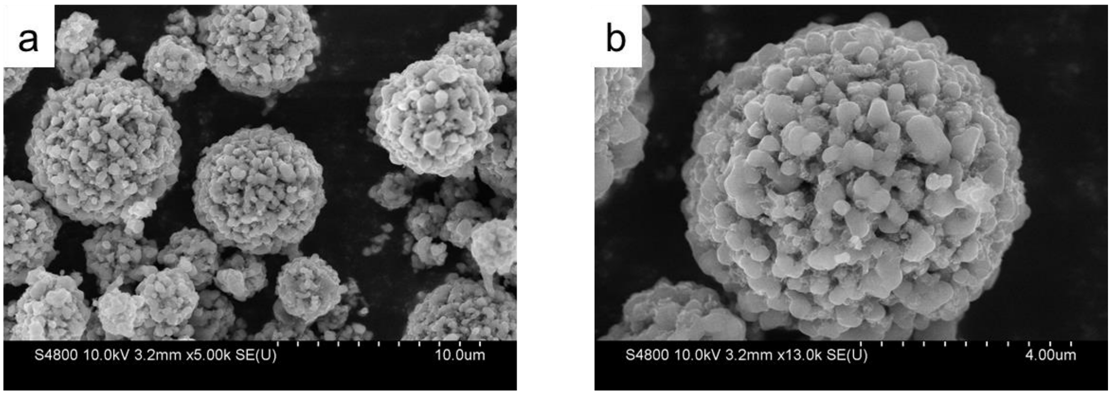

Supplementary Figure 18 | The SEM images of $LiVPO_4F$.

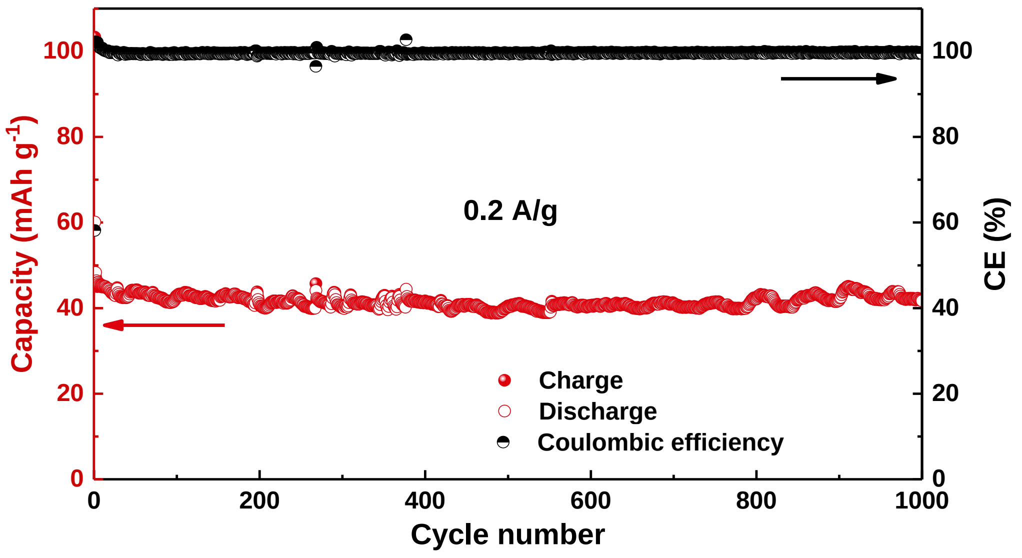

Supplementary Figure 19 | Cycling performance of LVPF that cycled in PPA electrolyte at a current density of 0.2 A g^{-1} at 25 °C.

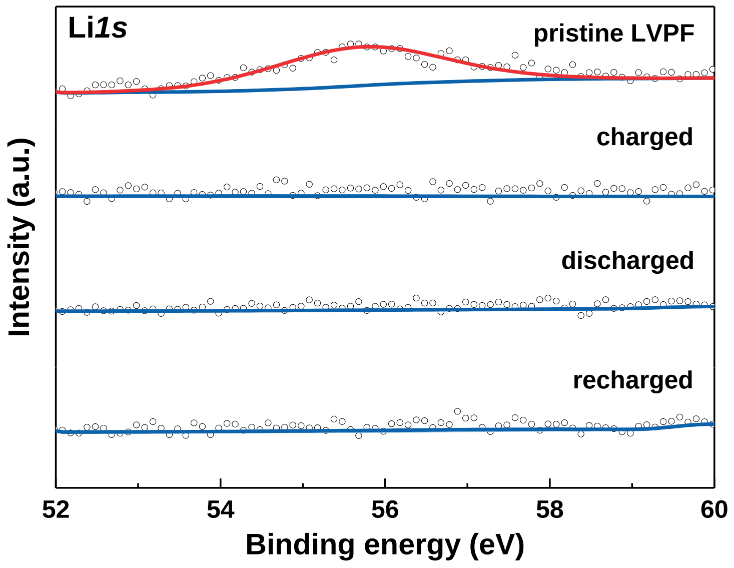

Supplementary Figure 20 | Li *1s* XPS spectra of $LiVPO_4F$ electrode at pristine, fully charged, fully discharged and recharged states.

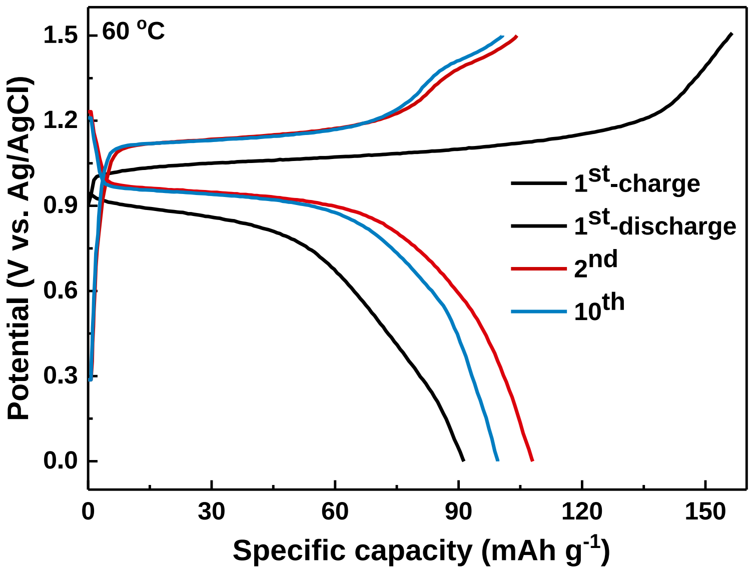

Supplementary Figure 21 | The voltage profile of a LVPF half-cell, whose electrolyte was replaced with new PPA after delithiation after the first charge process. The cell was tested at 60°C with a rate of 0.5 A/g.

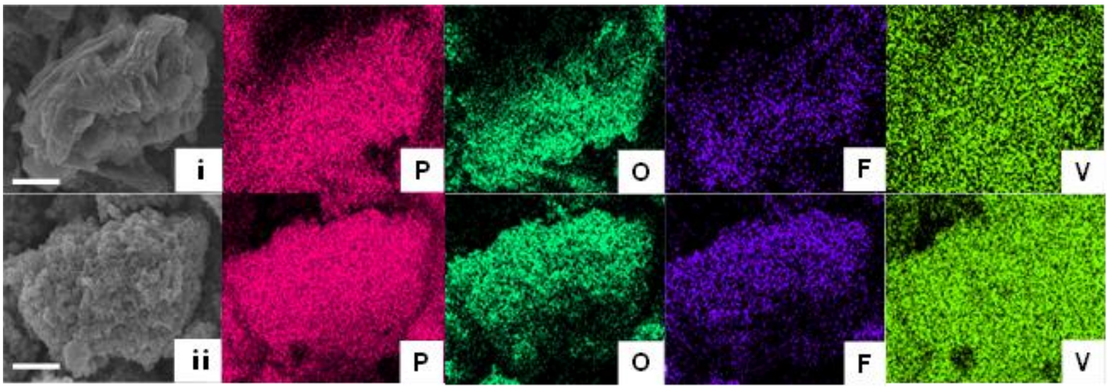

Supplementary Figure 22 | STEM EDS-elemental mapping images of elemental distributions on LVPF-electrodes at (i) charged and (ii) discharged states. Scale bar: 5 um.

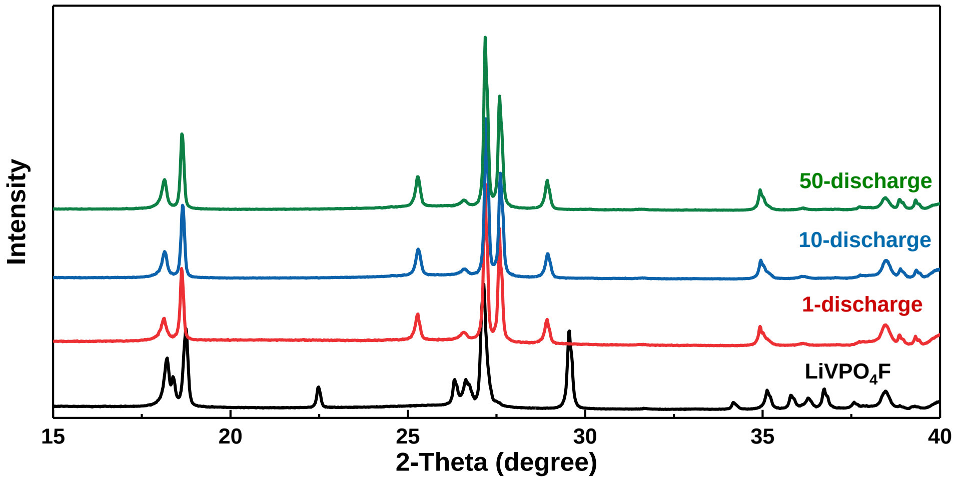

Supplementary Figure 23 | XRD patterns of the pristine $LiVPO_4F$ electrode (black), electrode cycling for 1 cycle (red), electrode cycling for 10 cycles (blue), and electrode cycling for 50 cycles (green).

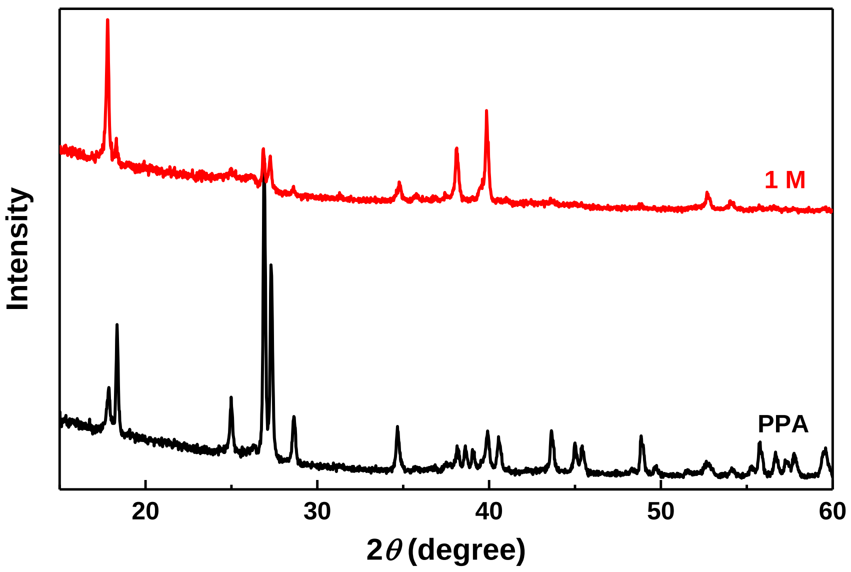

Supplementary Figure 24 | XRD patterns of the LVPF electrode cycled in PPA (black) or 1 M H_3PO_4 (red) for 10 cycles.

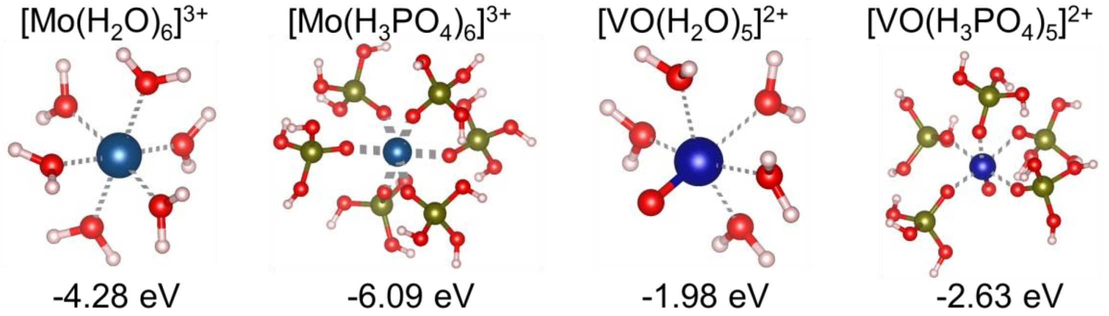

Supplementary Figure 25 | The quantum chemistry calculated solvation energies for Mo^{3+} and VO^{2+} in 1 M H_3PO_4 and PPA electrolyte. The negative values for the solvation energies indicate the energy favorable process. The results suggest that both Mo^{3+} and VO^{2+} will be dissolved into the electrolytes.

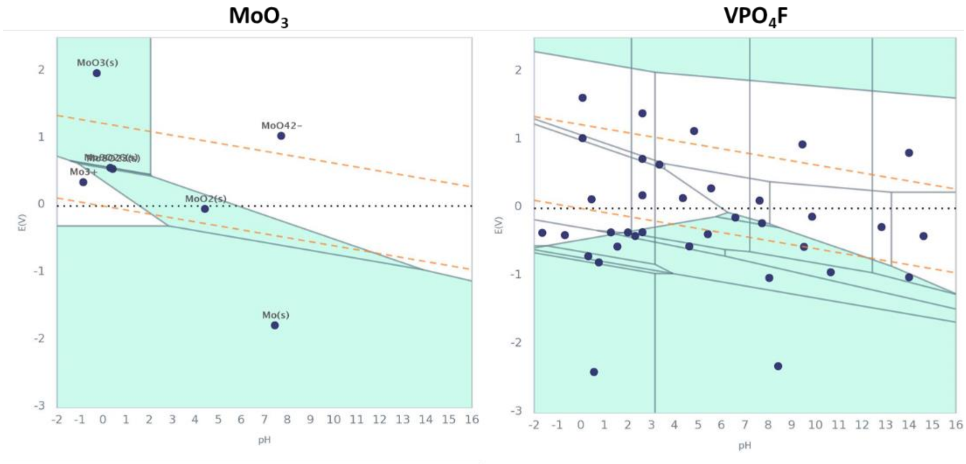

Supplementary Figure 26 | DFT predicted solid-aqueous equilibria for MoO_3 and VPO_4F. MoO_3 is stable with voltage >0.5 V versus standard hydrogen electrode (SHE) and PH<2. No solid VPO_4F can be stable in the aqueous solutions.

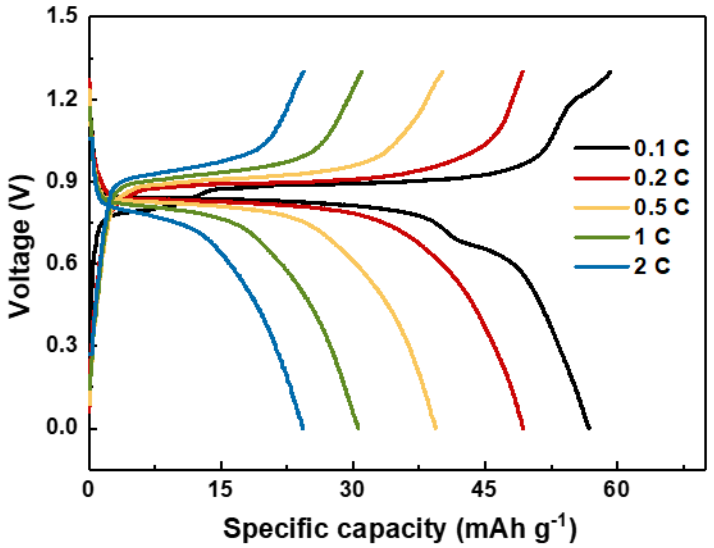

Supplementary Figure 27 | Rate performance of the proton full-cell, which was tested at 25℃.

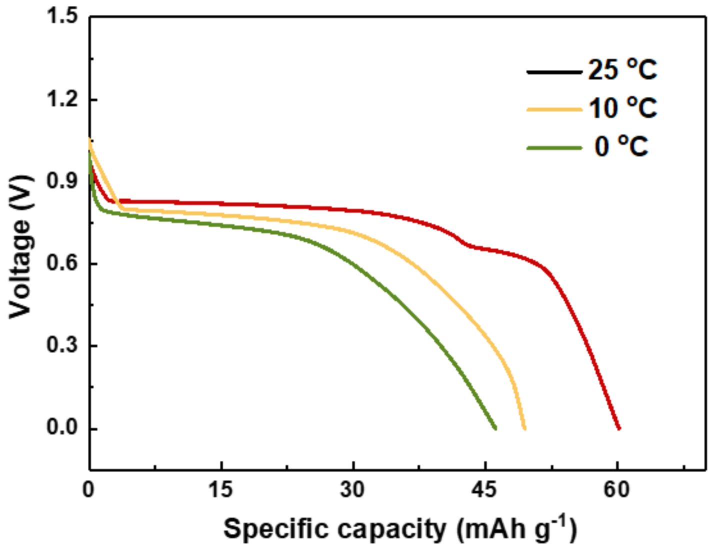

Supplementary Figure 28 | The discharge voltage profiles of the proton full-cell, which was tested at different temperatures with a rate of 0.1 C after charging at 60ºC.

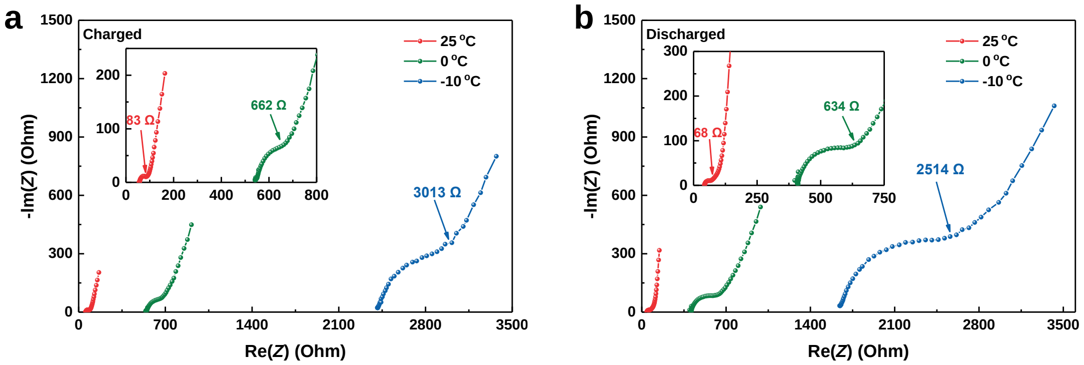

Supplementary Figure 29 | The Nyquist plots of MoO_3/$LiVPO_4F$ battery with charged or discharged states at different temperatures.

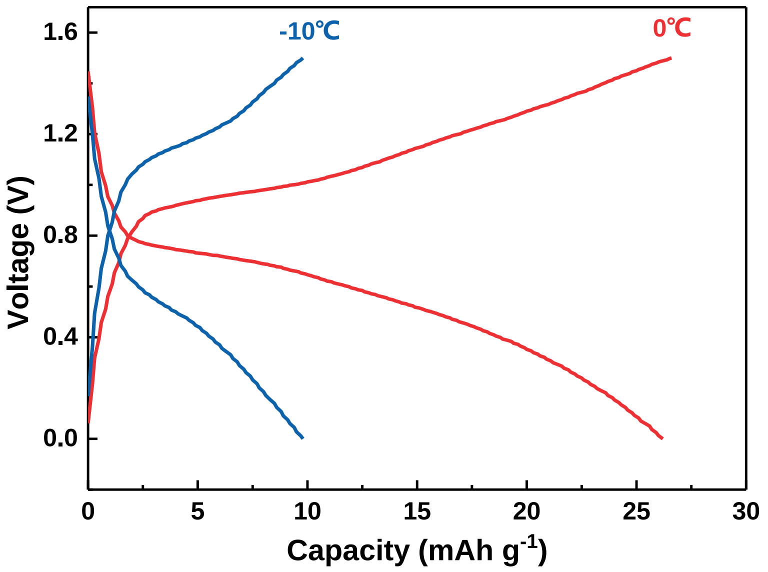

Supplementary Figure 30 | The voltage profiles of the proton full-cell cycled under low temperatures at a rate of 0.1 C.

Supplementary Table 1 | A comparison of the MoO_3//LVPF full cell with previously reported proton batteries, in terms of working temperature, specific capacity and energy density.

System	Electrolyte	Maximum specific capacity (mAh g^{-1})	Working temperature (°C)	Maximum energy density (Wh kg^{-1})
PTO//MnO_2@GF[4]	2 M H_2SO_4 + 2 M Mn_2SO_4	210 Based on anode	-70 ~ 25	132.6 Based on both
ALO//CF[5]	2 M HBF_4 + 2 M $Mn(BF_4)_2$	145.5 Based on anode	-90 ~ 25	132 Based on anode
Pb//PCHL-rGO[6]	5 M H_2SO_4	208 Based on cathode	-70 ~ 25	225 Based on cathode
MoO_3//MnO_2@GF[7]	2 M H_2SO_4 + 2 M Mn_2SO_4	209.6 Based on anode	-78 ~ 25	177.4 Based on both
MoO_3//CuFe-TBA[8]	$[Zn_3(H_2PO_4)_6(-H_2O)_3](BTA)$	55.4 Based on cathode	25 ~ 110	20 Based on cathode
TiO_2//MnO_2@CC[9]	0.5 M H_2SO_4 + 2 M Mn_2SO_4	106.5 Based on anode	25	143.2 Based on both
MoO_3//CuFe-TBA[10]	9.5 m H_3PO_4	50 Based on both	-88 ~ 25	40 Based on both
pDTP-AQ//pDTP-NQ[11]	1 M H_2SO_4	78 Based on both	25	31 Based on both
DHAQ//CF[12]	1 M H_2SO_4 + 0.5 M Mn_2SO_4	105 Based on anode	25	110 Based on anode
MoO_3//LVPF (This work)	PPA	85 Based on both	0 ~ 250	66.5 Based on both

Supplementary Note 1 | Calculations of the energy density and power density for the MoO_3//LVPF full cell.

The energy density of the MoO_3//LVPF full cell was calculated by following equation:

$$E = \frac{C*V}{m} \qquad \text{Equation (1)}$$

Herein, E is the energy density (Wh kg^{-1}); C is the discharge capacity (mAh); V is the average discharge voltage (V); m is the total mass (g) of anode (MoO_3) and cathode (LVPF).

The powder density of the MoO_3//LVPF full cell was calculated by following equation:

$$P = \frac{i*V}{m} \qquad \text{Equation (2)}$$

Herein, P is the power density (W kg^{-1}); i is the applied current (mA); V is the average discharge voltage (V); m is the total mass (g) of anode (MoO_3) and cathode (LVPF).

Supplementary References

1. Ines, B. et al. Synthesis, structure, and reactivity of a dihydrido borenium cation. *Angew. Chem. Int. Ed.* **50**, 8400-8403 (2011).
2. Kendall, R. A., Dunning, T. H. & Harrison, R. J. Electron affinities of the first-row atoms revisited. Systematic basis sets and wave functions. *J. Chem. Phys.* **96**, 6796-6806 (1992).
3. Lu, T. & Chen, F. Multiwfn: a multifunctional wavefunction analyzer. *J. Comput. Chem.* **33**, 580-592 (2012).
4. Guo, Z. et al. An organic/inorganic electrode-based hydronium-ion battery. *Nat. Commun.* **11**, 959 (2020).
5. Sun, T., Du, H., Zheng, S., Shi, J. & Tao, Z. High power and energy density aqueous proton battery operated at -90 °C. *Adv. Func. Mater.* **31**, 2010127 (2021).
6. Yue, F. et al. An ultralow temperature aqueous battery with proton chemistry. *Angew. Chem. Int. Ed.* **60**, 13882-13886 (2021).
7. Yan, L. et al. Solid-state proton battery operated at ultralow temperature. *ACS Energy Lett.* **5**, 685-691 (2020).
8. Ma, N., Kosasang, S., Yoshida, A. & Horike, S. Proton-conductive coordination polymer glass for solid-state anhydrous proton batteries. *Chem. Sci.* **12**, 5818-5824 (2021).
9. Geng, C. et al. Surface-induced desolvation of hydronium ion enables anatase TiO_2 as an efficient anode for proton batteries. *Nano Lett.* **21**, 7021-7029 (2021).
10. Jiang, H. et al. A high-rate aqueous proton battery delivering power below -78 °C via an unfrozen phosphoric acid. *Adv. Energy Mater.* **10**, 2000968 (2020).
11. Wang, X., Zhou, J. & Tang, W. Poly (dithieno[3,2-b:2',3'-d]pyrrole) twisting redox pendants enabling high current durability in all-organic proton battery. *Energy Storage Materials* **36**, 1-9 (2021).
12. Yu, J. et al. A crystalline dihydroxyanthraquinone anodic material for proton batteries. *Materials Today Energy* **22**, 100872 (2021).
